# New Yorkers

**Published:** 1868-04

**Authors:** 


### Hall's Journal of Health, April, 1868.

### NEW YORKERS.

There was an old woman who lived in a shoe, and had so many children, she did'nt know what to do ; still she had children and house, and both are very great blessings. I knew a man in early life who was rich, talented, famed and aristocratic; at the age of seventy years he wrote, "I am old and poor, and have no home." But he had souls for his hire, and I wish I was sure of having as glorious a home in heaven as I think he has now, for it is changeless and happy and holy, from which one goes no more out. But there are many who seem to want no permanent home; we have been told of New York ladies, who rather like the excitement of hunting a new home for every May moving, they certainly have a wide field for choosing, and have three months to make up their mind, as the time for offering houses begins on the first day of February, and it is not until daylight on the first of May following, that about half the families of New York City begin to "make a move." This custom of changing residences on the first day of May every year, or on any other one day, has its advantages in large cities ; and the experience of the old world seems to intimate, that altogether the advantages are greater than the discomforts. The moving in the French Metropolis is on the eighth and ninth of January ; on those days says a writer, the Parisians "may be seen going in families with their little, and often wretched collection of household goods piled in hand carts. The husband in the shafts of the vehicle, with a rope over his breast, pulls like a horse, the wife pushes behind, while children trot by the side. Generally, care and anxiety and want are imprinted on the face of the couple ; and even when this is not the case, the sight of the poor, ugly, dirty furniture seems to tell of misery endured. With snow in the streets and the fearful cold, the flitting family presents a spectacle peculiarly painful to witness." New York certainly has the advantage of Paris in having reasonably warm weather to move in, although it is pretty certain to rain on the first day of May in four cases out of five ; and it is one of the most tantalizing of rains ; if it would only rain a few hours and be done with it, or even if it were to pour down without ceasing from daylight to dark, there would be the advantage of knowing what to calculate upon ; but it never does that ; for there will be a delightful sunshine, and it often looks as if it did not intend to rain for a year, and you pile up your best furniture as long as you can find a hanging place on the vehicle, without any covering, when shortly after you have left the house, up comes a thundergust and down pours the rain, fast and furious, and so on with variations until night.

There are several " notions " among New Yorkers about selecting a dwelling. The streets cross Fifth Avenue at right angles, running east and west, beginning at Houston street which is really number one, and is one mile north of the Astor House, or one and a half miles north of Wall St. ; or one and three quarters of a mile from the Battery, which is the Southern limit of the city, being at the junction of the East River which separates it from Brooklyn, and the North or Hudson River, which divides it from Jersey City. So Fifth Avenue, begins at First, or Houston St., and runs nearly north to the entrance of Central Park, the distance being just three miles, presenting a length of palatial mansions not equalled in their princely grandeur in any city on the habitable globe. The houses on the streets which cross Fifth Avenue are numbered east and west ; the odd numbers being on the north side of the street ; the even, on the south side ; with this information, strangers can very easily find a dwelling, by going up Fifth Avenue until the number of the street is reached, and then turn east or west, that is right or left and walk until the number of the house is found. But in this arrangement there is one inconvenience ; if you want to go to 516 Fifth Avenue, you, as a stranger, do not know how far it is ; whether to walk or take a carriage, unless you know that on the avenues, twenty streets make a mile and that there are eight houses of full size of twenty-five feet front on each block, or between two streets. But the Philadelphia plan is about to be adopted which will simplify matters a great deal. There will be no

East and West street, no No. two west forty-third street, but it will become 502 Forty-third street, as the first house on first or Houston street and First Avenue will be No. one hundred and one, and so on until Second avenue is reached, when the first house west of Second Avenue will be Two hundred and one ; thus when you come to our present No. two, it will be Five Hundred and two, Forty Third Street, indicating that it is just west of Fifth Avenue ; the number of the avenue indicating the hundreth of the house. By the same rule, as the avenues begin at Houston or First street and run north, number one Fifth Avenue will become one hundred and one Fifth Avenue and the first house corner of forty-third street and fifth Avenue will be 4301 instead, as now 516, the first two figures indicate the number of the street, the remaining figures show the specific house in that block ; hence 4301 shows that you must come up to 43d street, and as twenty blocks or streets on the avenues make a mile, you must walk or ride two miles and one seventh. When you are in search of what is now No. two west 43d street, it will be five hundred and two, Forty-third street ; the first figure of the number indicating that it is near Avenue 5 or Fifth Avenue. But this is quite a digression, yet one of practical value to all strangers, and new to most New Yorkers. We were speaking of the queer notions some of our citizens had, and one of these is about the streets. Very many persons have an invincible repugnance to living in any street at any rental which has the name of " East " attached to it ; hence in reading the advertisements in the daily papers of persons who want to rent or purchase houses, an important specification is, "must be on the west side," that is west of Fifth Avenue. Thirty years ago, only the " nobodies " lived east of the Bowery. A house one hundred feet East of Fifth Avenue will not find as ready a sale or rental as its facsimile, one hundred feet west of Fifth Avenue. But the Area of New York City is so limited and so many persons of wealth, from all parts of the country are coming to the city to spend their money, and the remainder of their days, that the fashionable area is necessarily extended, so that persons are willing to go now as far east as Madison

Avenue, which is one block east of the Fifth ; nor are those who are "in Society," which means the upper ten ; the fashionable and rich, not the "new rich" but the old Knickerbockers, willing to go very far west, only a few doors west ; so that now the advertisements read, " must be between Madison and Sixth Avenues," they being the first streets on either side of the Fifth. But it is not all of Fifth Avenue, that is desired ground ; the lower portion of it is being turned into boarding houses, dress makers, millineries &c. And the turn things are now taking is indicated by the remark of a lady the other day, " no body cares to live below the forties," that is the richest and most aristocratic families were seeking residences " up town," that is above Fortieth street and it may give the reader some idea of what it costs to keep house on " The Avenue " above the " Forties," my neighbor and shrewd money making friend in a legitimate, honorable and useful business, on 42d street and the Avenue, was offered one hundred thousand dollars for his chaste and beautiful dwelling. and William H. Webb, the great ship-builder, who owns the opposite corner, would not take two hundred thousand for his magnificent mansion.

Within a few months, the house on Fifth Avenue, second north of Forty-third street, within two hundred feet of our door, same height, sold for one hundred and thirty-five thousand dollars cash ; and then had to be furnished at a cost of many thousand dollars besides, but it was within the charmed circle of Fifth Avenue and Murray Hill. To live on Fifth Avenue is great ; but to own a house in that limited part of Fifth Avenue called " Murray Hill," which begins on the south with the " Two Million " Palace of A. T. Stewart, and the mansions of the two Astors, and ends with " Chapin's Church on the north, the pinnacle of the hill being occupied by the " Brick Church," the oldest and most aristocratic and exclusive Presbyterian Church in New York, on the west side of the Avenue : the private residence of Governor Morgan and James Gordon Bennett of the New York Herald on the east side. Mr. Bennett's residence is said to be held at two hundred thousand dollars.

But it is a curious and instructive fact in connection with the habits of fashionable society, especially where it is rich, educated and refined, that there is substantial ground for their preferences, modes and observances ; this is strikingly so in the appropriation of Fifth Avenue for their homes ; it is on a ridge and the water runs from it, east and west, into the East and Hudson Rivers, consequently it must be the driest and healthiest street in New York, in its whole extent beginning with First or Houston street on the South, and ending at One hundred and fifty-fifth St, a distance of nearly eight miles ; but above Fifty-ninth St. at which is the entrance of the Central Park, which Fifth Avenue fronts for the distance of two miles and a half, there are no good dwellings ; on this line of two miles and a half the most fortunate, the richest of the rich will congregate within a very few years ; their dwellings on the east side of the avenue will face the Park, and their occupants will have all the advantages of the most beautiful public grounds in the world, without the expense of keeping them in order ; in summer they will hear the singing of the birds and look out upon the green pastures, where browse the gentle sheep and lambkins skip about in play and wide spreading trees cast their cooling shade reminding them ever of those fields of living green in Paradise beyond the Jordan of the grave into which they—can never come ! according to the generally received opinion. But my dear unsophisticated reader from the country, don't " lay this unction to your souls," we know that a good many of you would like to have it so. You have a kind of spite against the rich ; you feel that they have got more than their share of the good things of this life and you would rather be pleased to know, that on the other side they would have a little extra punching under the fifth ribs. But let us digress a little and talk about this matter a little bit. You may take it for granted as a very general rule, there are exceptions of course, that when a man is rich he has become so, by inheritance or by his own individual industry, sagacity, prudence and economy ; on the other hand the poor as a general rule are poor because they are deficient in industry, carefulness and self denial.

We went the other day to a shanty not large enough for a horse to live in, and certainly too filthy ; we wished to procure some fresh laid eggs for a special friend, an invalid lady who could not eat anything else with a relish ; the price was six cents each and they could have sold a bushel a day at that price, yet, although they were feeding a large number of hens, they had only two eggs to sell ; on enquiring the reason. "Ah yer honor the childther ate them all up." This simple incident shows the principle of the poverty of the poor. Here were children, dirty, ragged, without hats, bonnets, or shoes, eating eggs at a price which men worth a million would not pay for their own use, except in sickness. And yet if a rich man whose income amounts to hundreds of dollars a day, buys a dress for his wife or daughter at a hundred or two dollars, we snarl at "the extravgance of the rich." A thousand dollars for a dress would not be as striking an example of extravagance as these children in good health eating eggs at six cents a piece. But the egg belonged to the mother and she had a right to give it to her child ; the dress was paid for by the millionaire and he had a right to give it to his wife. Because a man is rich, shall we give him less liberty of action, than a city squatter in an eight by ten shanty? Surely we are coming to the days of "Squatter Sovreignty ;" the next step will be, to make kings of the poor, in virtue of their poverty ; and it is a fact, worthy of note that the newspaper press of New York, except those owned by men, who have become rich by their enterprize, do as a habit, inculcate and foster enmities, against the rich, as a class on the part of the masses. But look at the actual facts of the case. A man is not accounted "rich" in New York unless he is worth "clear" half a million of dollars ; but take all who are worth a hundred thousand dollars and over, men and women, and take an equal number of the poorest men and it will be found that the former are the best men in the city, the most christian, and the most liberal. We know a good methodist who not long ago gave forty thousand dollars to his church ; and another who gave five hundred thousand to establish a university for the education of young men to be circuit riders. We know a

Presbyterian Banker, whose income is said to be a thousand dollars a day, whose greatest delight is to give money, and not only money but his personal time and talents and labor to promote the better observance of the Sabbath; the suppression of intemperance; the abolition of gambling houses; the erection of churches; the establishment of schools for the poor; in short, there is nothing that promises to make men better and happier, that he does not engage in, heart in hand; another, an Episcopalian, who is never so happy as when he has a chance of helping somebody who needs and wants help. An old man was in my house within a year, he is in his grave now; he was a Quaker, he had retired from business, and was very rich. There was a benignity in his countenance and a majesty in his mein which showed at once that he was one of the excellent of the earth; he was all the time doing good, but no body knew it; just like those quiet Quakers; they interpret literally the Master's precept "Let not your left hand know what your right hand doeth." The widows at his funeral went away from his coffin saying, as the big tear-drops fell from their eyes, "He was the best friend we ever had." His way was to find out where the poor and needy lived; then if they merited assistance; next what they most needed; if it was a load of coal, the first thing they knew was a man at the door saying "a gintleman has sent ye a load of coal," or "I was directed to leave you a sack of flour." After he was gone, then these facts became known; although ofttimes the widow knew very well that "Mr. Walker sent it." Peace to the good man's memory! would there were ten to one like him.

Now let us tell you how many treat their preachers in New York city. One congregation was paying their minister four thousand dollars a year; but came to the conclusion to vote him another thousand. He wouldn't have it, saying, four thousand supplies all my reasonable wants and those of my family, I am not willing to receive it. They knew that it was not worth while to urge him; so they took the amount and had his life insured; in a year or two he died and the policy was paid over to his widow; but they concluded that was not enough; so they handed his widow twenty thousand dollars. I knew a minister who by a calamitous fire became involved for eight thousand dollars; one of the church said one day to the deacons, now friends its hard for our minister to have to pay for a dead horse; I'll pay half, if you will raise the other half; in an hour it was done; and on New Year's day following one of them on paying his New Year's visit, handed him the note cancelled. When his health failed him, and he was obliged to give up his church, they bought him a beautiful country place and some "five twenties," amounting in all to some twenty-five thousand dollars, to make him comfortable for the remainder of his days. I knew a man who had heard that a theological seminary needed some books; he went to the managers and said, "here are forty thousand dollars, use it to advantage." In the same year, the two modern "Cherryblee Brothers" heard that a large company of foreigners were worshipping in a church and were oppressed with a heavy mortgage, and they were poor; the next Sunday the minister announced that Mr. ——— had cancelled the mortgage and that the church was their own! in an instant the whole assembly of more than a thousand persons rose to their feet and bowed in silent reverence at the mention of their benefactor's name.

Said a lady to me the other day, I went to see my minister and thought he ought to take a recreation of six months abroad and to offer him the money to pay his expenses. It is now a custom in New York, if the minister breaks down to give him a holiday of two, ten or a dozen months, pay his expenses, hire a substitute and let him have his salary all the same. Havn't you heard of the clergyman who was disabled and on opening a new Bible presented to him by a friend he found a thousand dollar bill, and then turning another leaf there was another such bill and another and another until fifteen bills were found, but not the benefactor's name. Such is a specimen of the delicacy, generosity and magnanimity of the New York rich.

But it is seen in another direction; the care and respect and mindfulness of the churches towards their pastors, or rectors ; the arrangement between a minister and his people in New York is intended to be for life, and it is worthy of remark, that in conversing with the members of our principal churches, each is apt to consider his or her minister superior to any other ; and on his part, the minister makes the interest, the happiness and the welfare of his members identical with his own ; he ministers to them in sickness, he sympathizes with them in their sorrows ; and in their bereavements counsels, consoles and strengthens. When a clergyman in New York City once secures the confidence of his people, their generosity is worthy of all praise.

Not long ago, the church of which we have spoken as having dealt so liberally with its former pastors, now deceased or disabled, desired another and on the principle, that the sooner a vacancy is filled, the greater is the probability of a united choice, besides preventing a scattering of the flock, they determined promptly, with a unanimous will to invite a gentleman from abroad, who had preached to them once, a few weeks before. A cable despatch was sent to him, but nothing was said about the amount of salary. He promptly replied that he would come. On his arrival in October last, he was conducted to an appropriate residence, furnished from cellar to attic, with an ample supply of fuel and provisions; all the expenses for the removal of himself and family were reimbursed, with a salary proportionably liberal.

The readers of this Journal of Health! will no doubt wish to know what kind of a preacher is this for whom such handsome things are done and what kind of people are they who do such things, and so promptly too ; and what kind of preaching do they like. This may be answered by a correspondent of that excellent paper, the New York Examiner, who signs himself " A Baptist " who attended a recent ordinary service at the Presbyterian church in Fifth Avenue at Ninteenth street, of which Dr. John Hall, late of Dublin, is pastor. It will be read with interest :

"One Sunday afternoon, at a quarter past three o'clock, I went to a church on Fifth Avenue, not of our denomination. The house was crowded, people were standing in all the aisles, the galleries were full. Gentlemen had come early and taken their seats at the door of their pews in order to keep their pews for their families, knowing there would be a crowd. A very large proportion of the congregation was composed of young men and women. Seats were brought by the sexton and his assistants into the aisles, and were taken immediately, while numbers were compelled to stand during the whole service. And this in Fifth Avenue in the afternoon, when we are told good congregations cannot be gathered. A glance at the people showed that they had their homes in the immediate vicinity. It was not a congregation of fashionable people—but no one could avoid seeing that very many, perhaps most of those present, had long been accustomed to wealth.

I waited to find the solution of this Fifth Avenue riddle. The pastor entered the pulpit, and gave out a well known hymn. It was sung to a well known tune, a single chorister leading, and the whole congregation joining in the song. A chapter was read, followed by a simple, earnest prayer, another hymn was sung by all, and then the pastor read the first twenty verses of the first chapter of Isaiah, and preached to the people a running commentary on these verses.

There was no attempt at eloquence, no show of words. He began at that Scripture and preached unto them Jesus, explaining what the prophet said, showing its application to us at the present time, and pressing the application home upon the hearts of the people, not as rich or as poor, but as guilty sinners, lost, and needing the blood of Christ "so freely spilt for us."

The whole service and sermon were characterized by the greatest simplicity, and by a depth of feeling on the part of the pastor that was reciprocated by the audience. It was plain to all that the pastor's soul was on fire with love to Christ; that he longed with intense earnestness for the salvation of every one before him, and that he strove to set forth Jesus with all plainness of words so that none could miss his meaning.

What was it that drew that crowd out of their homes that lowering afternoon? It was not the expectation of hearing operatic music, nor to have their ears tickled by the orator's art, nor to wonder at the feats of an intellectual acrobat out of place, but it could only have been the desire to hear the pastor tell in loving tones the story of Jesus, and commend him to all. And there is more of this longing to hear of Jesus among all classes than we are apt to suppose, and God honors such preaching."

And let this suggestion go out on the wings of the wind to every priest and preacher in every clime and country and kindred, that the millionaire of "the Avenue," the slave of the "Plantation" and the "Ambassador of the olden time are alike drawn" with the cords of love, as with the bands of a man when they have "preached unto them Jesus ;" and that such preaching is "the wisdom of God and the power of God unto salvation ;" that there is something soothing in the description of a Saviour's love, whether it has been to the slave just from his hard task ; to the business man seeking rest from the harassments inseparable from worldly care or to ministers of state whose aching heads rest so uneasily even on downy pillows ; so truthfully has the poet written

"Jesus the name that calms our fears
That bids our sorrows cease
'Tis music in the sinners ears
'Tis life and joy and peace"

One of the strong points of Dr. Hall's preaching is, that he lures and soothes the sinner into the arms of the Savior ; for when he addresses him especially, he leans over the pulpit with both arms resting on it and hands clasped in front as if he would whisper confidingly in his ear, "Dear Brother, your character stands spotless before the world, by diligence and industry, by integrity and sagacity you have made yourselves rich, respected and loved ; and exercise a wide influence over the community ; will you not throw that influence on the side of your Savior by acknowledging him before men ; but if you remain aloof, others point at your high character as proof that men may be good out of the church, as well as in it, and thus you not only do not enter the kingdom yourself, but hinder those who might enter from coming in."

But what kind of men are they who, from hearing one or two sermons, had the sagacity to perceive that such a man would suit the people, whose representatives they were? They were bankers, retired merchants, presidents of monied institutions, and lawyers of ability—of high character and social position. It is the man who preaches Jesus unto them, who draws admiring crowds, filling every seat, crowding the aisles; and when the sexton, who always does everything at the right time and in the right way, has appropriated every seat, still they come; and well did one of the members say of the chosen man, "we have made a great Haul."

"T. L. C.," one of the ablest and most successful divines in this country, said of a speech of his on temperance: "He spoke with great simplicity and tenderness: it was a glorious speech, and strong as the Westminster Catechism. Truly, great honor is put on the name of HALL in these days." No doubt he was thinking, at the same time, of the Editor of HALL'S JOURNAL OF HEALTH, and that other greater and better man, NEWMAN HALL; and then, there is that other man of might, Dr. JOHN HALL, of Trenton, and Dr. JOHN HALL, President of Oxford University. Reader, please note, none of these are "kin to us;" we claim a royal line of descent from that "Big Injun," Pocahonty, all the way up through the Randolphs of Virginia, and the Halls of the Jersey sand flats, as the Book of American Heraldry fully sets forth.

It may be well to say here, that certain envious and ill-natured persons have said that "Pocahonty" wasn't much after all, but the facts of the case prove they are mistaken. Didn't she stick to "John Smith" through thick and thin, until he basely shook her off? Then didn't she turn round and stick to that other man? precisely as all the women do; when they take a notion to a fellow they hang on to the last gasp. For, as the Frenchman said, when he was admiringly describing the persistence of the Yankee character, "they have considerable *sticktion*."

The editor of the New York *Evening Mail*, which is so rapidly finding its way into every family of refinement and culture in this city, having heard one of Dr. Hall's sermons, said "it was superb." At each communion since his arrival, between twenty and thirty persons have joined his church, while his power over their pockets is as great as that over their hearts and christian affections; for after a sermon on foreign missions, one Sunday morning in January, a collection of $15,000 was taken; and the next Sabbath but one afterwards, for the promotion of the several Sunday schools of the congregation, $6,800 were put into the plates; such are the people, and such the minister, of whom a Methodist contemporary, of Boston, says:

"Cedar Street Church, where, half a century ago, the then distinguished Dr. Romeyn taught God's truth, two branches were cut off and planted up town. Over the one in University place, the stately, elegant and oratorical Dr. Potts was husbandman. The other, Dr. Alexander cultured, with his keen, bright pruning-knife. Both are considered as flourishing within the choice garden of metropolitan aristocracy; both are supposed to contain the *creme de la creme* of old Presbyterian piety.

In the footsteps of a pastor retiring with a well-filled purse from a generous people, comes Dr. John Hall, from Dublin. He finds a fine house elegantly furnished awaiting him, and a curious crowd rushing to hear him. Enter his church—a massive, brown-stone, high-spired edifice of Gothic architecture. Within, arch and architrave, column and plinth, painted in imitation of oak, waken in one a sort of druidical feeling for worship. In a deep recess, reaching to the roof and back of the pulpit, rises the grand organ; he who plays it is out of sight. There is no choir; the old-fashioned precentor is here revived; he stands at the minister's right, on a little lower platform, and starts the hymn in a clear, strong voice, and with one accord the whole people join in, making old Mear and Dundee swell up to the groined ceiling. Dressed with the conventional gown and bands, once rarely seen in the Presbyterian pulpits of America, but now fast growing common, the preacher, tall, stout, but with bended shoulders and somewhat ponderous movement, begins his service. His voice is strong and full, and his tone, though a little husky, is reverential. He reads with calm impressiveness; he prays with simple language, but without over-abounding fervor. There is no freshness, no mark of original genius in his utterances; rather does he seem purposely to avoid this and choose the language of God. The prayer is rather too long, which is perhaps unavoidable, as it is so comprehensive. The sermon comes; there is no rhetorical grace, no elocutionary effort, nothing startling or arousing. Occasionally the strong lion rises up, shakes himself, and turns around; but then he soon lies down again to a placid breathing."

